# Framing, Narratives, and the Overdose Crisis

**DOI:** 10.1017/jme.2025.10184

**Published:** 2025

**Authors:** Itai Bavli

**Affiliations:** School of Population and Public Health, The University of British Columbia, British Columbia, Canada

**Keywords:** Chronic pain, Opioid epidemic, Overdose, Stigma, Canadian Pain Task Force, Addiction

## Abstract

Narratives and frames have shaped the overdose crisis since its early stages. Efforts to control knowledge about the role of opioids in chronic pain have influenced clinical guidelines and prescribing behaviour. Dominant narratives shape policy by influencing how problems are defined, and which solutions are considered appropriate. A more nuanced understanding of how framing shapes interactions among stakeholders, including patients, clinicians, advocacy groups, industry, educators, and regulators, can clarify these dynamics. Engaging multiple perspectives, rather than relying on a single dominant narrative, offers a more effective path for addressing complex public health emergencies such as the overdose crisis.

In their recent article, Eisenkraft Klein et al.^1^ offer an important perspective on how the overdose crisis has been framed in Canada, focusing on the role of the Canadian Pain Task Force (CPTF) in shaping both the problem definition and the proposed solutions. They found that the Task Force framed the crisis primarily around limited access to opioids, emphasizing the harms of illicit drug use while downplaying the role of over-prescribing, and highlighting factors such as a lack of public and prescriber awareness and stigma toward people in pain. The proposed solutions focused on expanding access to prescription opioids and reducing stigma, rather than revisiting prescribing practices. These findings are important and warrant close evaluation.

Narratives and framing have shaped the overdose crisis from its early beginnings. Until the mid-1990s, physicians were generally reluctant to prescribe opioids for non-cancer chronic pain, due to well-established concerns about addiction and misuse. Recognizing a profitable market opportunity, Purdue Pharma, the maker of OxyContin, sought to change that narrative,^2^ employing a variety of tactics to influence healthcare professionals^3^ and increase opioid prescribing in both the United States (US)^4^ and Canada.^5^ Shaping, creating, controlling, and disseminating medical knowledge — often without the medical community being fully aware of these processes — has long been an effective marketing strategy for pharmaceutical companies.^6^ Understanding the importance of framing and narrative control, Purdue’s marketing strategy promoted opioids broadly — not just their blockbuster OxyContin. Other opioid manufacturers followed suit.

One of Purdue’s marketing goals was to reduce physicians’ “unjustified” fear of opioids, recognizing that this would best serve their commercial interests. In doing so, the company exploited the vulnerability of both patients suffering from inadequately treated pain and physicians who wanted to help them. In other words, Purdue understood that reshaping and controlling the narrative around opioid safety, such as promoting the idea that the risk of addiction and misuse was low, would help drive OxyContin sales. This framing proved effective. By the early 2000s, many physicians viewed opioids as safe and appropriate for chronic pain. Other companies, seeking to enter this profitable market, adopted similar promotional tactics, and prescribing rates soared.^7^

Framing opioids as safe and effective for chronic pain was also enabled by health regulators such as the Food and Drug Administration (FDA) and Health Canada (HC), who unintentionally contributed to this narrative by approving opioids for chronic pain — often in the absence of strong supporting evidence. For example, in 1995, the FDA stated on OxyContin’s label that the risk of addiction and misuse was low, and that the risk of misuse was lower than that of other, weaker opioids.^8^ Similarly, HC included information in OxyContin’s product monograph — approved just one month after the FDA’s decision in the US — suggesting that the risk of misuse was low.^9^ These claims were based on limited evidence,^10^ and Purdue’s sales team used them to further reinforce the narrative that slow-release formulations of opioids were safe to use.^11^

Other stakeholders, such as pain advocacy groups, which were often funded by opioid manufacturers,^12^ defended and helped amplify this narrative. Medical students were also introduced to inaccurate information about the true risks and benefits of opioids.^13^ These marketing and framing tactics not only promoted specific messages but also played a key role in reshaping the broader narrative around opioids. They influenced how the medical community and policymakers understood the appropriate use of these drugs, enabling opioid manufacturers to shape prescribing behaviours and influence clinical practice guidelines — contributing to widespread overprescription.

Eisenkraft Klein and colleagues demonstrate that framing and narrative remain as important today as they were in the past. They shape how stakeholders define a problem, influence which solutions are considered appropriate, and determine which are left out of the conversation, reinforcing particular policy directions. Their study encourages us to further investigate how framing shapes interactions among key actors, including patients, health professionals, medical societies, pharmaceutical companies, advocacy groups, and health regulators.

These findings are particularly important because CPTF stakeholders, unlike pharmaceutical companies, have no financial incentive. They understand better than most the daily struggles of people living with chronic pain and offer a view that must be taken seriously. Their responses reflect what they genuinely believe will best support those suffering from chronic pain, often grounded in personal experience as patients or caregivers. As a government-appointed advisory body composed of experts and advocates, the CPTF brings essential knowledge of the lived realities of pain, and their contribution to this discussion is both valuable and necessary.

However, this should not discourage us from finding the means to enable assessment of multiple alternative paths, such as the problem of addiction and drug misuse among pain patients, and the transition from prescription to street drugs. It is also important to review the evidence on the appropriate use of opioids, to further clarify what opioids are actually effective for,^14^ and to compare this with the views expressed by stakeholders. Gaining important knowledge from stakeholders should not deter us from discussing and debating alternative perspectives. The study’s findings should also encourage conversations about the possibility that one vulnerable group (pain patients) may unintentionally stigmatize another group (illicit drug users), raising the ethical question of what should be done to prevent it.

Framing and narrative can shape opioid policy and clinical guidelines by determining what is prioritized and what is left out of the conversation. They also shape how medical knowledge is produced and mobilized. This extends beyond opioid policy in Canada and encourages further examination and debate about the role of framing in health care.
